# Chlorate of Potassa in Typhoid Fever

**Published:** 1858-03

**Authors:** 


### Chlorate of Potassa in Typhoid Fever.

Whatever may be the value of the salt, heading this article, in the treatment of the disease indicated, (of which we are not competent to judge, from lack of experience, as well as from the utter uncertainty which overhangs in our minds all remedial agencies in this Fever,) our attention has been arrested by the somewhat striking coincidence of having been furnished with an article for this number of our Journal (which was in the printer's hands,) from a highly intelligent and reliable medical gentleman of this city, V. H. TALIAFERRO, M. D., lauding the Chlorate of Potassa in the treatment of Typhoid Fever, when we received the first number of the "Pacific Medical & Surgical Journal," containing a paper upon the same subject, by JAS. Morrison, M. D., of California, to which is subjoined a translation of an article from the *Gazette des Hospitaux*, on the "Treatment of Typhoid Fever with Chlorate of Potassa."

Though it has been a number of years since its first recommendation, if these gentlemen are not greatly mistaken in their conclusions, it has never received the attention deserved, and we would therefore commend the subject to the investigation of our readers. The article furnished from this city will be found under the Original head, and the other papers to which allusion has been made, we append.

*Treatment of Typhoid Fever with the Chlorate of Potassa.*

BY JAMES MORISON. M. D.

[Read before the Pacific Medical and Suigical Association, Dec. 17th, 1857.]

If he is to be regarded as "a public benefactor who makes two blades of grass grow where one grew before," certainly he is not less entitled to this distinction, who discovers and introduces a new remedial agent, possessing specific virtues, capable of resisting, and overcoming morbid action, or of neutralising and paralysing those inexplicable morbid agencies, which derange the whole animal economy, and, if uncontrolled, soon expend the powers of life.

Unfortunately for the credit of medical science, few medicines have continued, for a long time, to sustain the reputation, attending their first employment, while thousands are now regarded as inert or deleterious, which, if we can trust to the annals of medicine, have wrought almost miraculous cures, when administered by those who introduced them.

We have little sympathy with that tendency in the medical profession, for which it has been so oftan justly reproached, to follow in the beaten track, and reject every innovation without examination. We have, on the other hand, as little sympathy with those, who, disregarding the old axiom "in medio tutissimus ibis," rush into the other extreme, into the wild sea of empiricism, affecting to despise those sound principles established by the farther of medicine more than two thousand years ago, and which have been followed by the wisest of his succesors in the investigation and elucidation of medical truth. The Baconian system of philosophy did not originate with him whose name it bears. Hippocrates taught the principles of the inductive philosophy, at the bed-side of his patients, when he gave medical instruction to his pupils. He first observed, collected and classified, the phenomena of disease which he treated according to certain principles derived from observation and experience. It is only by the application of this system that any real advancement can be made in medical science, which has made such rapid progress within the last half century, when the great Napoleon declared "medicine to be a colleotion ot blind prescriptions whose result are more injurious than useful to humanity." Fifty years before, Cullen makes an admission not less flattering to the medical practice of his time. He says: "To me it appears probable that from the practice of medicine as it is at present exercised there arises much more mischief than advantage, and that it would, perhaps, be better for mankind if no medical art existed at all."

Isolated medical facts are of little importance. Obsevations and experiments must be upon an extensive scale to deduce from them any general laws or principles of practice. The innumerable medical fallacies and delusions which have been and are still in vogue, and which have brought the profession into disrepute, have had their origin in that tendency, which has continued to prevail, of deducing general principles from isolated facts. A remedy for example, has been given, an amelioration soon takes place in the condition of the patient and the medical agent receives the credit of effecting by its specific powers what has been, perhaps, only the result of the efforts of nature, of the "vis medicatrix nature. We cannot be too cautious in estimating the value of new remedies. They must be exhibited in many cases before their specific effects can be fully established.

How often do we find remedies ostentatiously introduced as specifics, and clamed as new, which have been long used by other members of the profession, with, perhaps, a satisfactory result, but without having discovered that they possess any remarkable specific qualities, and who are suddenly surprised that others should discover in them almost miraculous powers, which have so long escaped their own observation.

These observations have been suggested by an article in a late number of a French medical journal, the *Gazette des Hopitaux*, on the treatment of typhoid fever with the chlorate of potash. The author, Mr. Bellentani, formerly connected with the Hospitals of Orleans, claims for this agent, which has been long employed by other members of the profession, remarkable power in controlling and arresting the progress of this disease. This salt was formerly much used as a sedative and refrigerant in phthisis; and Dr. Stevens employed it in malignant fevers and cholera for promoting aterialization of the blood. The writer of this communication during his connection with the Baltimore Infirmary, as Resident Physician, from 1845 to 1849, frequently saw it administered for the treatment of typhoid fever by Prof Chew, of the University of Maryland, and with a success that will appear to confirm the bold and extravagant assertions of the French physician . In the year 1847, of seventy-two cases of typhoid fever which were treated in the Baltimore Infirmary only two had a fatal termination. It will be difficult to find, especially in the reports of Hospital practice, a more favorable result in the treatment of this grave disease. Prof. Chew's prescription was a tablespoonful of the following preparation every two hours, and this was continued until the commencement of convalescence: chlorate of potassa, one drachm; bicarb of soda and gum accacia, of each, two drachms; water, eight ounces.

While Mr. Bellentani is entitled to the credit of having brought this agent prominently before the public for the treatment of typhoid fever, it gives us pleasure to bear testimony to the fact that an American physician, whose "modesty is equalled only by his merits," has for at least twelve years employed the same remedy in the same disease with the most gratifying results.

With these preliminary observations we subjoin a translation of the entire article of Mr. Bellentani, which deserves the consideration of the medical profession. Even if it should appear on farther trial that this agent possesses no positive merits, it has, at least, the negative virtue of being a remedy which may be used with comparative safety in a disease over which medicines, hitherto, have exercised little control.

[*Treatment of Typhoid Fever with Chlorate of Potassa.* By MR. A. BELLENTANI, *ex-interne of the Hospitals of Orleans, etc.*]

The efforts of the practitioner should always be directed towards finding a specific to neutralise and curtail those diseases the first cause of which is unknown. Typhoid fever is one of those diseases. It is only by observations and experiments that we can discover the specific causes of diseases, and we are then led by induction to the choice of an agent capable of neutralizing it. Guided by these considerations, I have not hesitated to use in typhoid fever a new agent, so much and so justly vaunted in diphtheritic affections of the mouth. I refer to the chlorate of potassa. For the last six months I have employed it in all iorms of typhoid fever; thus far, with excellent effects; the disease has been controlled, its symptoms ameliorated, and convalesence established without delay. I give daily the following portion; gum water, two ounces; lemon syrup, one ounce and a half; chlorate of potassa, two scruples.

I increase the salt of potassa one scruple every other day. I have never given more than two drachms in twenty four hous. For drinks, acidulated infusions, or fresh water taken freely; a lavement of fresh water every day; applications to the abdomen of cold compresses, wet with the following solution: water, one quart; chlorate of potassa, one ounce; muriatic acid, two drachms and a half.

The portion is given every day, and continued until the symptoms amend; when convalescence is about to take place, then I discontinue all medication, and nourish the patient. I have treated by this means a great many typhoid cases; all have entirely recovered in a very short period. Convalescence began between the fifteenth, twentieth and thirtieth days; not in one case beyond the latter period.

